# Association Between the Use of Tobacco Products and Food Insecurity Among South Korean Adults

**DOI:** 10.3389/ijph.2022.1604866

**Published:** 2022-09-08

**Authors:** Seo Young Kang, Hong-Jun Cho

**Affiliations:** ^1^ International Healthcare Center, Asan Medical Center, Seoul, South Korea; ^2^ Department of Family Medicine, Asan Medical Center, University of Ulsan College of Medicine, Seoul, South Korea

**Keywords:** smoking, food insecurity, tobacco control, tobacco, tobacco products, South Korea

## Abstract

**Objectives:** Food insecurity is the most basic form of human deprivation; thus, strategies to eradicate poverty should include policies to improve food insecurity. This study investigated the association between the use of tobacco products and food insecurity.

**Methods:** We analyzed 21,063 adults from the Korea National Health and Nutrition Examination Survey, 2013–2015, 2019. The OR and 95% CI for food insecurity was calculated in each category of the status of tobacco products use and sociodemographic characteristics using multivariable logistic regression analysis.

**Results:** Of 21,063 participants, 7.3% belonged to the food insecurity group. The OR (95% CI) for food insecurity was 1.34 (1.08–1.65) among current users of any tobacco products compared with those who had never used any tobacco product. The odds for food insecurity were higher among those with secondhand smoke exposure, younger participants, those with lower household income, lower levels of education, manual workers or people without occupation, and separated/widowed/divorced participants.

**Conclusion:** Use of any tobacco products was associated with food insecurity among South Korean adults. Tobacco control could improve food insecurity.

## Introduction

Sustainable Development Goal 3 of the United Nations emphasizes ensuring healthy lives and promoting well-being for everyone [1]. Achieving food security is an important priority in this context because food, clothing, and shelter should be ensured for all people before considering healthcare as these are key to human survival. In fact, food insecurity is the most basic form of human deprivation and is addressed in any discussion on poverty [2] Food insecurity, the inability to consistently access enough food for a healthy life, implies a lack of financial resources for food at the household level [3]. It is associated with adverse health outcomes such as birth defects, anemia and asthma in children, and limitations in activities of daily living in older adults, as well as poverty [4]. Although ensuring food security alone would not alleviate poverty, strategies to eradicate poverty should include policies to improve food insecurity [2].

The use of tobacco products has nutritional implications. Cigarette smoking has been associated with food insecurity for adults and children in both high-income and low-income countries [5–7]. Although most studies investigating the relationship between smoking and food insecurity have a cross-sectional design [5–10], one prospective study among people living with HIV reported that cigarette smoking was an independent risk factor for food insecurity over the 12-month period [11]. Based on results of previous studies [5–11], several mechanisms have been proposed to explain the link between smoking and food insecurity. First, cigarette smoking may contribute to disproportionate spending of household income on cigarettes, thereby leading to food insecurity [12]. In the long run, smoking contributes to poor health, restricts workforce participation, and influences personal finances. Furthermore, nicotine addiction from smoking competes with household spending on food, ultimately resulting in food acquisition practices and dietary behaviours being altered [12]. Moreover, secondhand smoke (SHS) exposure increases the risks for diabetes, dyslipidemia, and metabolic syndrome in children via inflammatory effects [13]. In this context, Klein has proposed that tobacco control must be included as an environmental strategy of the nutrition agenda [14].

The use of new tobacco products such as electronic cigarettes (ECs) and heated tobacco products (HTPs) is dramatically increasing, resulting in significant public health concerns [15]. HTPs are tobacco products that generate an aerosol containing nicotine by heating the tobacco at a lower temperature than conventional cigarettes [16]. ECs and HTPs were released in the Korean market in 2007 and 2017, respectively. As of 2019, the current use of ECs and HTPs was reported as 5.1% and 8.8% among men and 1.4% and 1.5% among women in South Korea (hereafter Korea) [17]. Nevertheless, previous studies on the association between tobacco and food insecurity were mostly focused on conventional cigarette (CC) smoking. Therefore, in this study, we investigated the association of the use of all tobacco products with food insecurity using a nationwide representative sample of Korean adults. We hypothesized that the use of tobacco products would be positively associated with food insecurity.

## Methods

### Study Population

We used data from the Korea National Health and Nutrition Examination Survey (KNHANES) 2013 to 2015 and 2019. The KNHANES is a nationwide representative cross-sectional survey designed by the Korea Centers for Disease Control and Prevention (KCDC). The survey applies stratified, clustered, complex, and multistage probability sampling based on age, sex, and geographic areas. Further information about the study design and methodology has been reported previously [18]. The institutional review board of the KCDC approved procedures involving study participants, and all participants provided informed consent before participating in the survey. Of the 24,640 adults aged ≥19 years in the KNHANES 2013 to 2015 and 2019, 23,397 participants were valid as they had no missing values for strata, cluster, and sample weights for complex sampling analysis. Among them, we excluded those with missing responses for the questionnaires evaluating food insecurity (n = 2,334), leaving 21,063 participants for the final analysis.

### Use of Tobacco Products

The KNHANES is conducted annually, and the survey items are slightly different each year. With regard to the survey items evaluating the use of tobacco products, the KNHANES 2013 and 2014 contain items evaluating the use of CCs and ECs. The KNHANES 2015 contains items evaluating the use of CCs, ECs, snus, hookah, cigars, and other tobacco products, and the KNHANES 2019 contains items evaluating the use of CCs, ECs, HTPs, snus, hookah, cigars, and other tobacco products.

CC smoking was evaluated by the following two questions: “How many cigarettes have you smoked in your lifetime?” Response options were either “< 100 cigarettes,” “≥ 100 cigarettes,” or “I’ve never smoked cigarettes.” Participants who chose “< 100 cigarettes” or “≥ 100 cigarettes” were asked to answer the next question: “Do you currently smoke cigarettes?” Response options were either “I smoke cigarettes every day,” “I occasionally smoke cigarettes,” or “I smoked cigarettes in the past but not now.” Based on the above questions, participants were classified into never CC smokers, former CC smokers, and current CC smokers. EC use was evaluated by the following two questions: “Have you ever used ECs?” (yes/no). Participants who responded “yes” were asked to answer the next question: “Have you ever used EC in the past 1 month?” (yes/no). Based on the above questions, participants were categorized into ever EC users, former EC users, and current EC users. HTP use was evaluated by the following two questions: “Have you ever used HTPs (eg. IQOS, glo, lil, etc.)?” (yes/no). Those who responded “yes” were asked to answer the next question: “Do you currently use HTPs (eg. IQOS, glo, lil, etc.)?” Response options were either “I use HTPs every day,” “I occasionally use HTPs,” or “I used HTPs in the past but not now.” Based on the above questions, participants were classified into never HTP users, former HTP users, and current HTP users. Participants were additionally asked to select all tobacco products that they have ever used and products that they have used in the past 1 month from the following response options: snus, hookah, cigar, others, and none. From these responses, we determined never use, former use, and current use of snus, hookah, cigar, and other tobacco products.

Based on the above survey items, participants were classified as never users of any tobacco products, former users of any tobacco products, or current users of any tobacco products.

### Definition of Food Insecurity

Household food security status was evaluated using an 18-item questionnaire developed for Koreans based on the United States Household Food Security Survey Module (US HFSSM) [19]. The 18-item questionnaire evaluates dietary experience resulting from a lack of money during the past year. Among the 18 items, 8 items were targeted towards households with children. Therefore, households with children responded to all 18 items, whereas households without children responded to 10 items. The sum of the food security scores was calculated based on the responses to each item of the questionnaire. Food security status was categorized into the following four groups according to the food security scores: food secure group (score 0–2), food insecure group without hunger (score 3–7 for household with children, score 3–5 for household without children), moderate food insecure group with hunger (score 8–12 for household with children, score 6–8 for household without children), and severe food insecure group with hunger (score 13–18 for household with children, score 9–10 for household without children). Food insecure group with hunger means where people report reduced quality or variety of diet without reduced food intake. Moderate food insecure group with hunger indicates when people have uncertainty about the ability to obtain food due to insufficient money or resources. People in this group may have skipped meals or run out of food occasionally. Severe food insecure group with hunger means when people run out of food due to financial deprivation and may spend an entire day without eating. Food insecure group without hunger, moderate food insecure group with hunger, and severe food insecure group with hunger were grouped into the food insecure group.

### Covariates

We collected sociodemographic variables including age, sex, household income, educational levels, occupation, and marital status. Age was divided into the following four categories: 19–34 years, 35–49 years, 50–64 years, and ≥65 years. Household income was divided into quartiles and then categorized as low (first quartile), middle (second and third quartiles), and high (fourth quartile). Educational levels were categorized into: less than middle school education, middle school graduate, high school graduate, and college graduate. Occupation was initially divided into the following 10 categories: managers, professionals, clerks, service, sales, skilled agriculture/forestry/fishery, craft/trades, equipment-machine operating, and assembly, elementary, and armed forces according to the Korean Standard Classification of Occupations [20]. Managers, professionals, and clerks were further categorized as non-manual workers; the rest were categorized as manual workers. Therefore, participants were classified into either non-manual workers, manual workers, or no occupation. Marital status was categorized into either married/cohabitating, separated/widowed/divorced, or unmarried.

Participants’ alcohol consumption status was categorized according to the definitions of the National Institute on Alcohol Abuse and Alcoholism [21]. For men aged <65 years, heavy drinking was defined as > 14 standard glasses per week, and moderate drinking was defined as 1–14 standard glasses per week. For men aged ≥65 years or women aged <65 years, heavy drinking was defined as > 7 standard glasses per weak, and moderate drinking was defined as 1–7 standard glasses per week. For women aged ≥65 years, heavy drinking was defined as > 3 standard glasses per weak, and moderate drinking was defined as 1–3 standard glasses per week. Finally, alcohol consumption was categorized into either non-drinker, moderate drinker, or heavy drinker.

SHS exposure at home as a proxy for another family members’ tobacco product use was evaluated by the following question: “In the past 7 days, have you inhaled other people’s cigarette smoke indoors in your home?” Participants who answered “yes” were considered to have SHS exposure at home; whereas, participants who answered “no” or “No one in the family regularly smokes cigarettes indoors at home” were considered not having SHS exposure at home.

### Statistical Analysis

Analyses were conducted after accounting for the complex sample design and the sample weights. We performed the Chi-square test to compare the characteristics of the participants in the food secure group and in the food insecure group. We performed multivariable logistic regression analysis to evaluate the association between the use of tobacco products and food insecurity. The odds ratios (ORs) and 95% confidence intervals (CIs) for food insecurity was calculated in each category of the status of tobacco products use and sociodemographic characteristic after adjusting for age, sex, household income, education, occupation, marital status, alcohol consumption, SHS exposure at home, and use of tobacco products. Furthermore, we calculated the ORs and 95% CIs for different levels of food insecurity compared with the food secure group in each category of the status of tobacco products use in order to evaluate the presence of a dose-response relationship. Analyses were performed with IBM SPSS Statistics for Windows version 23.0 (IBM Corp., Armonk, NY, USA), and two-tailed *p* values <0.05 were considered statistically significant.

## Results

### Comparison of the Characteristics Between Food Secure Group and Food Insecure Group

Table 1 shows the sociodemographic characteristics and the use of tobacco products according to food security status. Of the 21,063 participants, 92.7% (n = 1,598) belonged to the food secure group and 7.3% belonged to food insecure group. Specifically, 6.1% (n = 1,328) belonged to the food insecure group without hunger, 1.1% (n = 246) belonged to the moderate food insecure group with hunger, and 0.1% (n = 24) belonged to the severe food insecure group with hunger (not shown). The proportions of participants with food insecurity increased as age increased, were higher among females than among males, and increased as household income and educational levels decreased. With regard to occupation, the proportion of participants with food insecurity was highest among those without occupation, and also were higher among manual workers than among non-manual workers. Furthermore, the proportions of participants with food insecurity were highest among separated/widowed/divorced participants, and higher among unmarried participants than among married/cohabitating participants. The proportions of participants with food security were higher among non-drinkers than among moderate or heavy drinkers, and higher among current users of any tobacco products than among never or former users of any tobacco products.

**TABLE 1:** Characteristics according to food security status (*n* = 21,063) (Korea. 2013–2015 and 2019).

	Food secure (*n* = 19,465)	Food insecure (*n* = 1,598)	Odds for food insecurity
	N (%)	N (%)	OR (95% CI)
Age (years)			
19–34	3,676 (93.5)	254 (6.5)	1.00
35–49	5,279 (93.2)	362 (6.8)	1.04 (0.86–1.26)
50–64	5,488 (93.1)	417 (6.9)	1.05 (0.87–1.28)
≥65	5,022 (89.6)	565 (10.4)	1.66 (1.37–2.01)
Sex			
Male	8,334 (93.4)	608 (6.6)	1.00
Female	11,131 (92.0)	990 (8.0)	1.23 (1.10–1.37)
Household income			
High	5,822 (99.0)	55 (1.0)	1.00
Middle	10,136 (92.7)	746 (7.3)	7.92 (5.04–12.45)
Low	3,413 (80.2)	781 (19.8)	25.01 (15.87–39.42)
Education			
College graduate	6,311 (96.7)	206 (3.3)	1.00
High school graduate	5,985 (92.4)	462 (7.6)	2.40 (1.93–2.98)
Middle school graduate	1,807 (89.8)	183 (10.2)	3.34 (2.58–4.32)
Less than middle school	3,819 (86.4)	571 (13.6)	4.64 (3.77–5.71)
Occupation			
Non-manual	4,243 (96.9)	132 (3.1)	1.00
Manual	6,388 (92.3)	522 (7.7)	2.61 (2.11–3.23)
No occupation	7,284 (90.6)	772 (9.4)	3.23 (2.62–3.98)
Marital Status			
Married/Cohabitating	13,920 (94.2)	857 (5.8)	1.00
Separated/Widowed/Divorced	2,568 (83.3)	502 (16.7)	3.25 (2.76–3.83)
Unmarried	2,947 (92.9)	234 (7.1)	1.24 (1.03–1.48)
Alcohol consumption			
Non-drinker	5,321 (90.0)	591 (10.0)	1.00
Moderate drinker	10,222 (94.0)	662 (6.0)	0.58 (0.50–0.66)
Heavy drinker	3,000 (92.7)	237 (7.3)	0.72 (0.59–0.87)
^a^Use of tobacco products			
Never use of any tobacco product	11,327 (93.1)	854 (6.9)	1.00
Former use of any tobacco product	3,846 (93.6)	278 (6.4)	0.93 (0.79–1.08)
Current use of any tobacco product	3,351 (91.3)	344 (8.7)	1.28 (1.09–1.49)
Secondhand smoke exposure at home			
No	17,068 (93.1)	1,316 (6.9)	1
Yes	1,458 (89.6)	172 (10.4)	1.56 (1.27–1.92)

a Tobacco products in 2013 and 2014: conventional cigarettes (CCs) and electronic cigarettes (ECs).

Tobacco products in 2015: CCs, ECs, snus, hookah, cigars, and other tobacco products; Tobacco products in 2019: CCs, ECs, heated tobacco products, snus, hookah, cigars, and other tobacco products.

OR, odds ratio; CI, confidence interval.

Values are presented as unweighted numbers (weighted percentages).

### Factors Associated With Food Insecurity

Table 2 shows the multivariable analysis results for the factors associated with food insecurity. Compared with those who had never used any tobacco product, the odds for food insecurity were higher among the current users of any tobacco products. In the multivariable model, the OR (95% CI) for food insecurity was 1.34 (1.08–1.65) among current users of any tobacco products and 1.16 (0.95–1.41) among former users of any tobacco products. Furthermore, the odds for food insecurity were higher among those with SHS exposure at home (OR 1.32, 95% CI 1.06–1.65) than those without SHS exposure at home. The odds for food insecurity were lower among older participants (OR 0.55, 95% CI 0.40–0.76 for those aged 50–64 years; OR 0.27, 95% CI 0.18–0.39 for those aged ≥65 years compared with 19–34 years), higher among those with lower household income (OR 6.60, 95% CI 4.08–10.65 for middle income; OR 18.21, 95% CI 11.02–30.10 for low income compared with high income), and higher among those with lower levels of education (OR 1.69, 95% CI 1.34–2.13 for high school graduate; OR 2.47, 95% CI 1.79–3.41 for middle school graduate, OR 3.11, 95% CI 2.29–4.24 for less than middle school education compared with college graduate). Furthermore, the odds for food insecurity were higher among manual workers (OR 1.27, 95% CI 1.00–1.62) and among those with no occupation (OR 1.48, 95% CI 1.17–1.88) compared with non-manual workers, and higher among separated/widowed/divorced participants (OR 1.85, 95% CI 1.53–2.23) compared with married/cohabitating participants. Compared with non-drinkers, the odds for food insecurity were lower among moderate drinkers (OR 0.75, 95% CI 0.64–0.88). Regression analysis results with 0.01, and 0.001 significance levels are presented in Supplementary Tables S1, S2.

**TABLE 2:** Multivariable analysis for the factors associated with food insecurity (Korea. 2013–2015 and 2019).

	^a^OR (95% CI)
^b^Use of tobacco products	
Never use of any tobacco product	1.00
Former use of any tobacco product	1.16 (0.95–1.41)
Current use of any tobacco product	1.34 (1.08–1.65)
Secondhand smoke exposure at home	
No	1.00
Yes	1.32 (1.06–1.65)
Age (years)	
19–34	1.00
35–49	1.07 (0.83–1.38)
50–64	0.55 (0.40–0.76)
≥65	0.27 (0.18–0.39)
Sex	
Male	1.00
Female	1.08 (0.91–1.28)
Household income	
High	1.00
Middle	6.60 (4.08–10.65)
Low	18.21 (11.02–30.10)
Education	
College graduate	1.00
High school graduate	1.69 (1.34–2.13)
Middle school graduate	2.47 (1.79–3.41)
Less than middle school	3.11 (2.29–4.24)
Occupation	
Non-manual	1.00
Manual	1.27 (1.00–1.62)
No occupation	1.48 (1.17–1.88)
Marital Status	
Married/Cohabitating	1.00
Separated/Widowed/Divorced	1.85 (1.53–2.23)
Unmarried	1.09 (0.83–1.43)
Alcohol consumption	
Non-drinker	1.00
Moderate drinker	0.75 (0.64–0.88)
Heavy drinker	0.82 (0.64–1.04)

a Adjusted for age, sex, household income, education, occupation, marital status, alcohol consumption, secondhand smoke exposure, and use of tobacco products.

b Tobacco products in 2013 and 2014: conventional cigarettes (CCs) and electronic cigarettes (ECs).

Tobacco products in 2015: CCs, ECs, snus, hookah, cigars, and other tobacco products.

Tobacco products in 2019: CCs, ECs, heated tobacco products, snus, hookah, cigars, and other tobacco products.

OR, odds ratio; CI, confidence interval.

Table 3 shows the association between use of tobacco products and different levels of food insecurity. The OR (95% CI) for food insecurity without hunger, moderate food insecurity with hunger, and severe food insecurity with hunger were 1.28 (1.03–1.60), 1.48 (0.84–2.58), and 7.82 (2.11–28.94), respectively among current users of any tobacco products. The odds increased as the levels of food insecurity worsened (p for trend <0.05). A dose-response relationship between former use of tobacco products and different levels of food insecurity was not observed.

**TABLE 3:** Association between use of tobacco products and different levels of food insecurity (Korea. 2013–2015 and 2019).

	Food insecure group without hunger vs. food secure group	Moderate food insecure group with hunger vs. food secure group	Severe food insecure group with hunger vs. food secure group	P for trend
	^a^OR (95% CI)	^a^OR (95% CI)	^a^OR (95% CI)	
^b^Use of tobacco products				
Never use of any tobacco product	1.00	1.00	1.00	
Former use of any tobacco product	1.15 (0.94–1.40)	1.17 (0.61–2.23)	2.66 (0.58–12.28)	0.085
Current use of any tobacco product	1.28 (1.03–1.60)	1.48 (0.84–2.58)	7.82 (2.11–28.94)	0.002

a Adjusted for age, sex, household income, education, occupation, marital status, alcohol consumption, and secondhand smoke exposure.

b Tobacco products in 2013 and 2014: conventional cigarettes (CCs) and electronic cigarettes (ECs).

Tobacco products in 2015: CCs, ECs, snus, hookah, cigars, and other tobacco products.

Tobacco products in 2019: CCs, ECs, heated tobacco products, snus, hookah, cigars, and other tobacco products.

OR, odds ratio; CI, confidence interval.

## Discussion

In this study, current use of any tobacco product was associated with food insecurity. Furthermore, dose-response relationship was observed between current use of any tobacco products and food insecurity levels. Other factors associated with food insecurity were SHS exposure at home, lower household income, lower levels of education, being manual workers or having no occupation, and being separated, widowed, or divorced. Conversely, older people and moderate drinkers were less likely to be food-insecure.

Previous studies have shown that cigarette smoking creates or exacerbates financial burden by competing with spending on daily necessities [9–12, 22]. In one study, daily tobacco use was associated with higher odds for severe food insecurity, whereas nondaily tobacco use was not associated with food insecurity [10]. This may suggest that frequent tobacco use, or more spending on tobacco, can aggravate food insecurity. In one longitudinal study, cigarette smoking at baseline was associated with greater levels of food insecurity at 12 months by negatively affecting one’s health status and restricting workforce participation [11]. This prospective study emphasized a unidirectional relationship between smoking and food insecurity. Furthermore, due to smoking-induced deprivation and financial stress, smoking and nonsmoking households will eventually have different food acquisition practices and dietary behaviors [5, 23]. The dose-response relationship between current use of tobacco products and food insecurity levels in our study further supports the view that higher levels of smoking-induced financial deprivation results in aggravation of food insecurity. Moreover, the positive association between SHS exposure and food insecurity in our analysis shows that spending on tobacco products by other family members may worsen food insecurity because family members often share household income as well as food. On the flip side, stress and psychological distress caused by food insecurity may reinforce smoking behavior and prevent smoking cessation [12, 24]. Nicotine, a component of tobacco, is an appetite suppressant [25]. People with food insecurity could smoke cigarettes as a way to control their hunger. As our study showed a positive association between food insecurity and the use of any tobacco products, strategies which have proven to be effective in reducing smoking, such as increases in tobacco price, mass media campaigns, and comprehensive smoke-free policies, should be applied to all types of tobacco products. The resulting drop in household spending on tobacco products will increase resources available for other household necessities, including food; thus, these strategies would eventually improve food security.

Numerous studies have shown a positive association between substance use including alcohol consumption and food insecurity [26–29], while one study reported that food insecurity had a protective effect on hazardous drinking [30]. Studies reporting a positive association between drinking and food insecurity explain that disproportionate spending of income on alcohol may lead to food insecurity [26–29]. Contrastingly, a protective effect of drinking on food insecurity could possibly indicate that drinkers generally have more discretionary income [31]. In this study, alcohol use seems to be protective of food insecurity. We are not sure whether this association is true or due to unmeasured confounders. Further study is needed to evaluate the effect of alcohol consumption on food insecurity.

Consistent with previous study findings, lower household income, lower levels of education, and being manual workers or having no occupation were associated food insecurity in this study [32, 33]. Furthermore, higher levels of food insecurity among separated, widowed, or divorced participants indicate the need for ensuring food security in these groups of people in Korea. In the crude analysis, older age was associated with higher levels of food insecurity; however, this association was reversed in the multivariable analysis after adjusting for multiple socioeconomic covariates. Food security has been relatively ensured among older adults in Korea. In fact, the prevalence of food insecurity in Korea is relatively low compared with other countries. According to the World Bank data, the prevalence of moderate or severe food insecurity in Korea was 5.1% in 2019, which was much lower than the global prevalence of 27.6% [34]. Many districts in Korea have provided community health promotion projects, which include nutritional support for older people and those with socioeconomic disadvantages [35]. These projects may have contributed to the low prevalence of food insecurity in Korea.

In this study, socioeconomically disadvantaged populations such as those with lower income, lower educational levels, and manual workers or those with no occupation were more likely to experience food insecurity. Therefore, policies to improve food security in Korea should target these populations. Policies for food security can be classified as follows: policies for food demand, policies for access through agricultural markets, and policies for supply [36]. Further, improving socioeconomic disparities is a good strategy to improve food security because food availability depends heavily on people’s income, assets, and entitlements and improving food access and supply does not always guarantee food security for everyone [36]. Furthermore, evidence based tobacco control for all types of tobacco products is needed as current use of any tobacco products as well as SHS exposure were associated with food insecurity in Korea.

There are several limitations in this study. First, due to the cross-sectional nature of the study design, we were unable to draw the cause and effect relationship between the use of tobacco products and food insecurity. It would be meaningful to conduct longitudinal studies on this association as the bidirectional relationship between CC smoking and food insecurity has been reported previously. Second, the types of tobacco products included each year in the KNHANES were different. However, HTPs became available in Korea from June 2017, and the proportions of participants using snus, hookah, cigars, and other tobacco products were very small; thus, the overall results would not have been influenced. Third, we were not able to evaluate the smoking status of family members although it could be a potential confounder for the association between tobacco products use and food insecurity. Instead, we evaluated SHS exposure of the study participants at home, which could explain the smoking status of other household members.

In conclusion, current use of any tobacco products was associated with food insecurity. Evidence-based tobacco control policies could improve food security and reduce poverty.
