# Supplementary material for: Association Between the Use of Tobacco Products and Food Insecurity Among South Korean Adults
Source: Int J Public Health. 2022 Sep 8;67:1604866. doi: 10.3389/ijph.2022.1604866 (PMC9492844; doi:10.3389/ijph.2022.1604866)
Supplement: Supplementary file 1 [file DataSheet1.docx]

**Supplementary Table 1** Odds ratios for food insecurity in each variable with different significance levels (Korea. 2013-2015 and 2019)

|  | OR (99% CI) | OR (99.9% CI) |
| --- | --- | --- |
| Age (years) |  |  |
| 19-34 | 1.00 | 1.00 |
| 35-49 | 1.04 (0.80-1.34) | 1.04 (0.75-1.44) |
| 50-64 | 1.05 (0.82-1.36) | 1.05 (0.77-1.45) |
| ≥ 65 | 1.66 (1.28-2.14) | 1.66 (1.20-2.30) |
| Sex |  |  |
| Male | 1.00 | 1.00 |
| Female | 1.23 (1.07-1.42) | 1.23 (1.02-1.48) |
| Household income |  |  |
| High | 1.00 | 1.00 |
| Middle | 7.92 (4.37-14.36) | 7.92 (3.70-16.96) |
| Low | 25.01 (13.74-45.50) | 25.01 (11.63-53.78) |
| Education |  |  |
| College graduate | 1.00 | 1.00 |
| High school graduate | 2.40 (1.81-3.19) | 2.40 (1.67-3.46) |
| Middle school graduate | 3.34 (2.38-4.69) | 3.34 (2.16-5.16) |
| Less than middle school | 4.64 (3.53-6.10) | 4.64 (3.27-6.58) |
| Occupation |  |  |
| Non-manual | 1.00 | 1.00 |
| Manual | 2.61 (1.97-3.46) | 2.61 (1.82-3.74) |
| No occupation | 3.23 (2.46-4.24) | 3.23 (2.28-4.58) |
| Marital Status |  |  |
| Married/Cohabitating | 1.00 | 1.00 |
| Separated/Widowed/Divorced | 3.25 (2.63-4.03) | 3.25 (2.47-4.27) |
| Unmarried | 1.24 (0.98-1.57) | 1.24 (0.92-1.67) |
| Alcohol consumption |  |  |
| Non-drinker | 1.00 | 1.00 |
| Moderate drinker | 0.58 (0.48-0.69) | 0.58 (0.46-0.73) |
| Heavy drinker | 0.72 (0.55-0.93) | 0.72 (0.51-1.00) |
| ^*^Use of tobacco products |  |  |
| Never use of any tobacco product | 1.00 | 1.00 |
| Former use of any tobacco product | 0.93 (0.76-1.13) | 0.93 (0.71-1.20) |
| Current use of any tobacco product | 1.28 (1.04-1.56) | 1.28 (0.98-1.66) |
| Secondhand smoke exposure at home |  |  |
| No | 1 | 1 |
| Yes | 1.56 (1.19-2.05) | 1.56 (1.10-2.21) |

OR: odds ratio, CI: confidence interval

^*^Tobacco products in 2013 and 2014: conventional cigarettes (CCs) and electronic cigarettes (ECs);

Tobacco products in 2015: CCs, ECs, snus, hookah, cigars, and other tobacco products;

Tobacco products in 2019: CCs, ECs, heated tobacco products, snus, hookah, cigars, and other tobacco products

**Supplementary Table 2** Multivariable analysis for the factors associated with food insecurity with different significance levels (Korea. 2013-2015 and 2019)

|  | ^†^OR (99% CI) | ^†^OR (99.9% CI) |
| --- | --- | --- |
| ^*^Use of tobacco products |  |  |
| Never use of any tobacco product | 1.00 | 1.00 |
| Former use of any tobacco product | 1.16 (0.89-1.50) | 1.16 (0.83-1.61) |
| Current use of any tobacco product | 1.34 (1.01-1.76) | 1.34 (0.94-1.90) |
| Secondhand smoke exposure at home |  |  |
| No | 1.00 | 1.00 |
| Yes | 1.32 (0.99-1.77) | 1.32 (0.91-1.92) |
| Age (years) |  |  |
| 19-34 | 1.00 | 1.00 |
| 35-49 | 1.07 (0.76-1.49) | 1.07 (0.70-1.64) |
| 50-64 | 0.55 (0.36-0.84) | 0.55 (0.32-0.95) |
| ≥ 65 | 0.27 (0.16-0.44) | 0.27 (0.14-0.50) |
| Sex |  |  |
| Male | 1.00 | 1.00 |
| Female | 1.08 (0.86-1.35) | 1.08 (0.81-1.44) |
| Household income |  |  |
| High | 1.00 | 1.00 |
| Middle | 6.60 (3.51-12.39) | 6.60 (2.94-14.78) |
| Low | 18.21 (9.41-35.27) | 18.21 (7.82-42.42) |
| Education |  |  |
| College graduate | 1.00 | 1.00 |
| High school graduate | 1.69 (1.25-2.29) | 1.69 (1.14-2.49) |
| Middle school graduate | 2.47 (1.62-3.78) | 2.47 (1.44-4.25) |
| Less than middle school | 3.11 (2.07-4.67) | 3.11 (1.85-5.23) |
| Occupation |  |  |
| Non-manual | 1.00 | 1.00 |
| Manual | 1.27 (0.93-1.75) | 1.27 (0.85-1.91) |
| No occupation | 1.48 (1.08-2.03) | 1.48 (0.99-2.21) |
| Marital Status |  |  |
| Married/Cohabitating | 1.00 | 1.00 |
| Separated/Widowed/Divorced | 1.85 (1.44-2.36) | 1.85 (1.35-2.53) |
| Unmarried | 1.09 (0.76-1.56) | 1.09 (0.69-1.72) |
| Alcohol consumption |  |  |
| Non-drinker | 1.00 | 1.00 |
| Moderate drinker | 0.75 (0.61-0.93) | 0.75 (0.57-0.99) |
| Heavy drinker | 0.82 (0.59-1.13) | 0.82 (0.54-1.23) |

OR: odds ratio, CI: confidence interval

^*^Tobacco products in 2013 and 2014: conventional cigarettes (CCs) and electronic cigarettes (ECs);

Tobacco products in 2015: CCs, ECs, snus, hookah, cigars, and other tobacco products;

Tobacco products in 2019: CCs, ECs, heated tobacco products, snus, hookah, cigars, and other tobacco products

^†^Adjusted for age, sex, household income, education, occupation, marital status, alcohol consumption, secondhand smoke exposure, and use of tobacco products
